# Host serine protease ACOT2 assists DENV proliferation by hydrolyzing viral polyproteins

**DOI:** 10.1128/msystems.00973-23

**Published:** 2023-12-19

**Authors:** Sen Ma, Sai Shi, Binghong Xu, Meijun Liu, Lei Xie, Yang Su, Jiachen Li, Qinqin Liang, Sheng Ye, Yaxin Wang

**Affiliations:** 1 Frontiers Science Center for Synthetic Biology (Ministry of Education), Tianjin Key Laboratory of Function and Application of Biological Macromolecular Structures, School of Life Sciences, Tianjin University, Tianjin, China; 2 Center of Basic Molecular Science, Department of Chemistry, Tsinghua University, Beijing, China; National Institute of Allergy and Infectious Diseases (NIAID), National Institutes of Health (NIH), Bethesda, Maryland, USA

**Keywords:** ABPP, DENV, ACOT2, serine protease, virus replication

## Abstract

Dengue fever is a mosquito-borne tropical disease caused by the dengue virus (DENV). The replication of DENV relies on the processing of its genome-encoded polyprotein by both viral protease NS3 (NS3^pro^) and host proteases. However, the impact of host proteases on DENV proliferation is not well understood. In this study, we utilized fluorophosphonate-based probes (FPs) to investigate the up-regulation of host serine proteases during DENV infection in detail. Among the identified proteases, acyl-CoA thioesterase 2 (ACOT2), an enzyme that hydrolyzes acyl-CoA molecules to generate fatty acids and free CoA, exhibited cleavage activity against DENV polypeptide substrates. Enzymatic assays and virological experiments confirmed that ACOT2 contributes to DENV propagation during the replication stage by cleaving the viral polyprotein. Docking models provided insights into the binding pocket of viral polypeptides and the catalytic mechanism of ACOT2. Notably, this study is the first to demonstrate that ACOT2 functions as a serine protease to hydrolyze protein substrates. These findings offer novel insights into DENV infection, host response, as well as the potential development of innovative antiviral strategies.

**IMPORTANCE:**

DENV, one of the major pathogens of Dengue fever, remains a significant public health concern in tropical and subtropical regions worldwide. How DENV efficiently hijacks the host and accesses its life cycle with delicate interaction remains to be elucidated. Here, we deconvoluted that the host protease ACOT2 assists the DENV replication and characterized the ACOT2 as a serine protease involved in the hydrolysis of the DENV polypeptide substrate. Our results not only further the understanding of the DENV life cycle but also provide a possibility for the usage of activity-based proteomics to reveal host-virus interactions.

## INTRODUCTION

Dengue fever is a prevalent mosquito-borne arboviral infection that is endemic in urban and suburban areas of tropical and subtropical countries worldwide (1). Globally, there are approximately 390 million reported cases of dengue virus infections each year, with nearly 96 million individuals exhibiting clinical symptoms such as dengue hemorrhagic fever (DHF) and dengue shock syndrome (DSS) (2). The pathogen dengue virus (DENV) is primarily transmitted by mosquito vectors, predominant members of the species Aedes aegypti and Aedes albopictus (3). DENV consists of four distinct serotypes (DENV 1–4), which are classified based on the antigenicity of the viral envelope protein (4). Among these serotypes, DENV-2 is known to cause the most severe cases of DHF and DSS, for which effective targeted therapies are still lacking (5).

DENV belongs to the *Flavivirus* genus of the *Flaviviridae* family and possesses a single-stranded, positive-sense RNA genome of approximately 11 kb (6). The genome encodes an open reading frame (ORF) that is translated into a polyprotein. Both viral and host proteases process the polyprotein into three structural proteins [Capsid (C), Membrane (M), and Envelope (E)] and seven nonstructural proteins (NS1, NS2A, NS2B, NS3, NS4A, NS4B, and NS5) (7). Among these proteins, the viral NS3 protein is a chymotrypsin-like serine protease that requires the cofactor NS2B for activation. NS2B/NS3 protease (NS2B/NS3^pro^) is responsible for cleaving the viral polyprotein precursor and plays a critical role in DENV replication (8–10). However, apart from the furin protease, which cleaves pre-Membrane protein (prM) during virus maturation (11), little is known about the cellular proteases involved in DENV replication.

The proteolytic cleavage of the viral polyprotein by virus and cellular proteases is crucial for the viral reproduction of progeny (12). Therefore, a direct quantification method is required to study the specific type of protease in complex proteomes. Activity-based protein profiling (ABPP) is a functional proteomic technology that utilizes chemical probes, with the majority active site-directed, to label and monitor the functional status of proteases *in vitro* and *in vivo* (13–15). The basic unit of ABPP is the probe, which contains a reactive warhead that selectively binds and covalently labels catalytically active proteases, a reporter tag that is used for detection, enrichment, identification, and a binding group for optimizing the binding affinity or cell permeability (16). Over the past decade, ABPPs have proven to be valuable chemical proteomics tools for investigating the role of enzymes in viral infection. For example, the DCG-04 probe was used to study the role of cellular cathepsin L in SARS-CoV and Ebola virus infections (17, 18), while probes containing a β-lactam warhead were employed to demonstrate increased FASN activity during HCV replication (19).

In this study, we aimed to investigate the involvement of host proteases, particularly serine proteases, in DENV viral proliferation. Serine proteases are among the largest and most diverse enzymes in host cells (20), and identifying these cellular serine proteases and characterizing their functions is crucial for understanding virus-host interaction, molecular pathogenesis, and the development of antiviral strategies. To achieve this, we utilized a fluorophosphonate (FP) probe that covalently binds to serine residues of cellular proteins in complex proteomes during DENV infection. Through this approach, we identified a series of up-regulated serine proteases, with acyl-CoA thioesterases 2 (ACOT2) displaying the most pronounced hydrolytic activity on the viral polyprotein. Previous studies have shown that ACOT2 catalyzes the hydrolysis of CoA esters to free CoA and carboxylic acids, playing essential roles in lipid metabolism and other cellular processes (21). However, its function as a serine hydrolase in promoting viral replication by hydrolyzing the DENV viral polyprotein has never been reported. These findings not only uncover novel functions of host proteins but also elucidate their significant roles in DENV virus propagation, thereby providing potential pharmaceutical targets for the development of antiviral drugs.

## RESULTS

### Selection and optimization of activity-based serine probe

**Fig 1:**
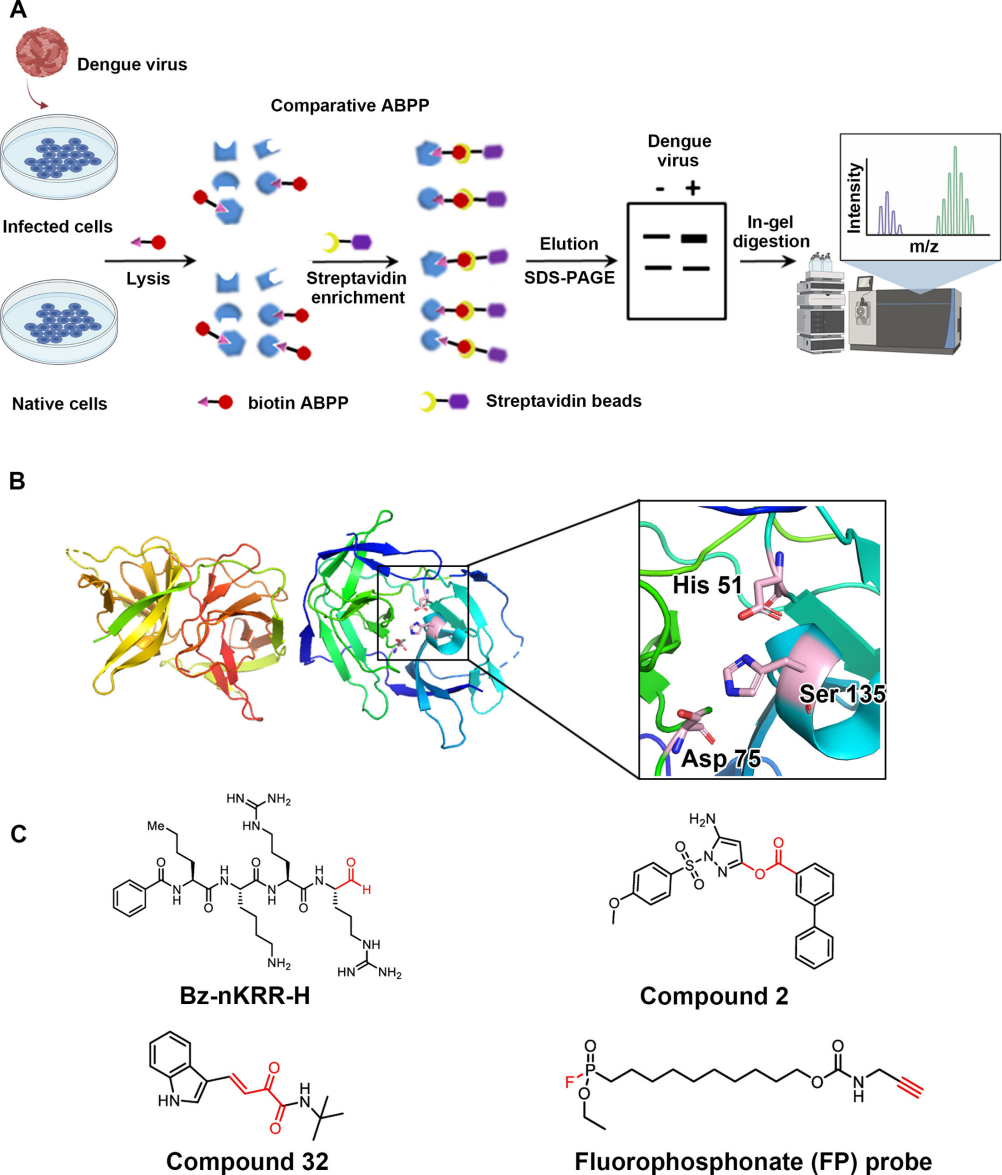
Selection of activity-based serine ABP probe. (**A**) Scheme of comparative ABPP to identify proteins. (**B**) Structure of DENV viral protein NS2B/NS3^pro^ and its catalytic triad His51-Asp75-Ser135. (**C**) Inhibitors of NS2B/NS3^pro^ and fluorophosphonate probe.

In this study, we employed streptavidin beads to enrich serine proteins labeled with an activity-based probe (ABP) by conjugating the probe to biotin-azide using copper-catalyzed azide-alkyne cycloaddition (CuAAC) (22). The candidate proteins were separated by SDS-PAGE and subjected to label-free quantification by LC-MS/MS following in-gel trypsin digestion (Fig. 1A). By comparing the proteomic changes between DENV-infected and -uninfected cells, we identified the target proteins of interest.

The initial and crucial step involved selecting an effective ABP with a specific affinity for labeling the target serine protein superfamily in complex proteomes. One effective approach to designing the probe is based on covalent inhibitors of the target protein. In a previous study, we designed ABPs using rupintrivir and NK-1.8k, which are covalent inhibitors of EV71 3C^pro^, to investigate host factors in response to polypeptide hydrolysis and replication during EV71 infection (23). Therefore, we gave priority to choosing inhibitors that covalently bind to the DENV NS2B/NS3^pro^ serine protease as the reactive group for designing our ABPs. The NS2B/NS3^pro^ active center consists of a catalytic triad formed by His51-Asp75-Ser135 (Fig. 1B). Through a literature survey, we identified Bz-nKRR-H and α-ketoamide-β, γ-unsaturated ester (compound 32) as compounds that form reversible covalent bonds with Ser135 of NS2B/NS3^pro^. In addition, Compound 2, a DENV protease inhibitor based on pyrazole ester derivatives, can covalently bind to Ser135 of NS2B/NS3^pro^ (Fig. 1C). However, attaching reporter groups through biorthogonal reactions proved challenging. Due to these limitations, we were unable to improve the serine ABPs based on existing covalent inhibitors. Therefore, we utilized the traditional fluorophosphonate (FP) probe, which targets the serine hydrolase superfamily, for comparative ABPP to investigate changes in host serine proteins after DENV infection (Fig. 1C; Fig. S1) (24).

To achieve optimal labeling efficiency, we first determined the DENV multiplicity of infection (MOI) and infection time to A549 cells. We utilized the expression of Renilla luciferase to reflect the DENV multiplication (25). The results showed that the DENV infection A549 cells after 72 hours released the most strong fluorescence signal and then decreased rapidly, which was the same phenomenon at different MOIs of DENV. We then carried out an analysis of A549 cell lysates that were infected with DENV at an MOI of 5 and 72 hours post-infection (hpi) as a background to explore the labeling efficiency of FP. TAMRA-azide-tagged FP was used to optimize the concentration of FP and the reaction time by gel fluorescence detection (Fig. S2B and S2D). To avoid the nonspecific labeling by a high concentration of FP and the instability of the host cells’ proteome, we chose FP with stronger signals at a concentration of 50 µM after 30 minutes of incubation as the final reaction conditions (Fig. S2D and S2E).

### Targets identification using comparative ABPP

Comparative ABPP was conducted to identify the cellular serine proteins in response to DENV infection. A549 cells were either infected with DENV at MOI of 5 or left uninfected and were harvested at 72 hours post-infection (72 hpi). Cell lysates were treated with an FP for 30 minutes, followed by the addition of Biotin-N_3_, which reacted with the alkynyl group of the FP *via* CuAAC. The labeled proteins were then enriched using streptavidin beads, separated by SDS-PAGE, and subjected to LC-MS/MS analysis with label-free quantification after in-gel trypsin digestion. To minimize variations, we performed three independent replicate experiments and considered proteins that were identified in at least two experiments as candidates.

**Fig 2:**
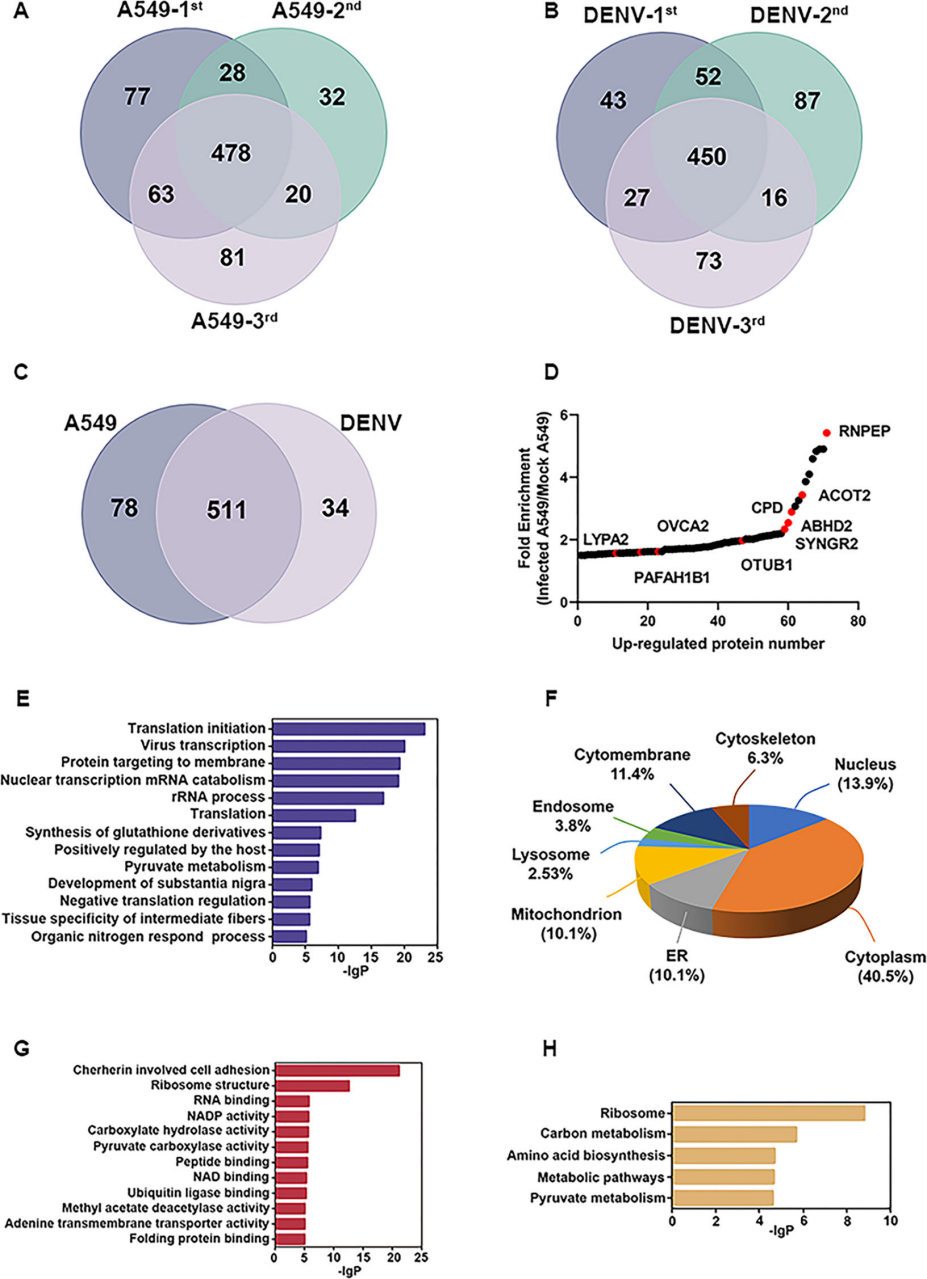
Target identification by comparative ABPP. (**A**) Targets identified in native A549 cells (A549-1^st^, A549-2^nd^, and A549-3^rd^ represent the A549 cell lysates assay from three biologically independent experiments). (**B**) Targets identified in DENV-infected A549 cells (DENV-1^st^, DENV-2^nd^, and DENV-3^rd^ represent DENV-infected A549 cell lysates assay from three biologically independent experiments). (**C**) Overlap of the proteins identified in A549 cells and DENV-infected A549 cells. (**D**) Representative ratio plot for up-regulated proteins identified by FP from the active proteome infected versus mock. Red points indicate the up-regulated host proteases with serine residues. (**E**) Biological processes analysis of up-regulated proteins identified by FP. (**F**) Cellular component analysis of up-regulated proteins. (**G**) GO analysis of molecular functions. (**H**) KEGG analysis of target proteins. ER, endoplasmic reticulum.

In the comparative ABPP assays, we identified 589 and 545 cellular proteins, at least characterized by two biological experiments, in DENV-uninfected and -infected A549 cell lysates, respectively (Fig. 2A and B). To ensure specificity, we cross-checked the identified targets from both groups and obtained a total of 511 common targets (Fig. 2C). Among these common targets, 71 proteins were discovered to be up-regulated by more than 1.5-fold, and Gene Ontology (GO) analysis showed that the up-regulated proteins identified by FP were distributed in intracellular compartments (Fig. 2D and F). The analysis of biological processes and molecular functions revealed that the targets identified in the comparative ABPP were predominantly involved in crucial processes related to virus infection, such as virus transcription, translation, and RNA catabolism (Fig. 2E and G). Furthermore, the Kyoto Encyclopedia of Genes and Genomes (KEGG) analysis indicated that these proteins are associated with various pathways involved in biosynthetic and metabolic (Fig. 2H).

### Identification of upregulated host serine proteases

Nine-host proteases with FP-labeled serine residues were discovered in up-regulated proteins by comparative ABPP analysis. These proteases include Monoacylglycerol Lipase Abhydrolase Domain Containing 2 (ABHD2), Acyl-Coenzyme A Thioesterase 2 (ACOT2), Carboxypeptidase D (CPD), Lysophospholipase 2 (LYPA2), Ubiquitin Thioesterase OTU Domain (OTUB1), Esterase Ovarian Tumor Suppressor Candidate 2 (OVCA2), Platelet-Activating Factor Acetylhydrolase 1B Subunit (PAFAH1B1), Aminopeptidase B (RNPEP) and Synaptogyrin-2 (SYNGR2) (Fig. 2D; Table S1).

**Fig 3:**
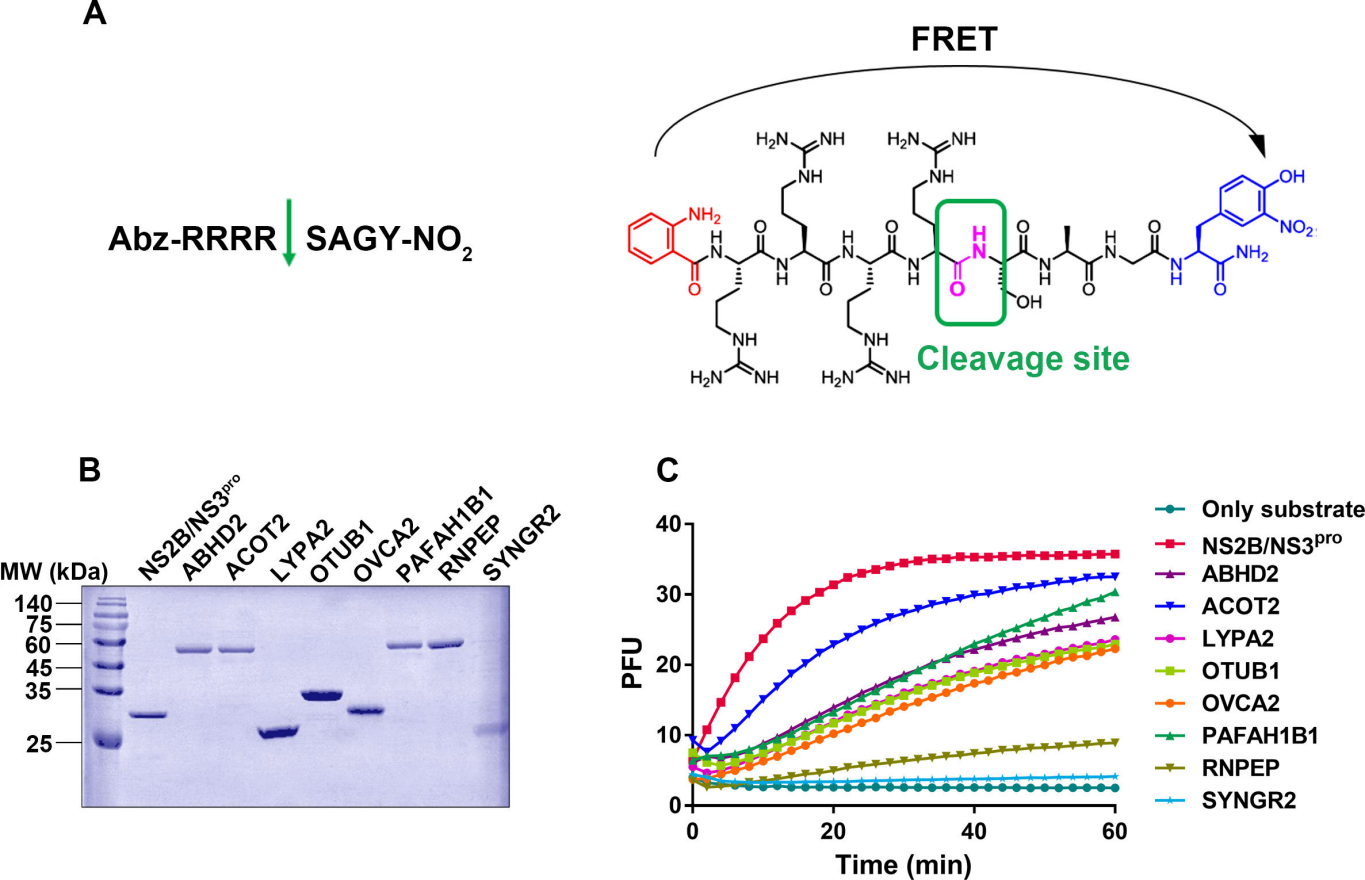
The hydrolytic activities of target proteins on the substrate of DENV NS2B/NS3^pro^. (**A**) The structure of fluorophore peptide substrate for NS2B/NS3^pro^. (**B**) SDS-PAGE analysis of the expression and purification for up-regulate host protease. (**C**) The fluorophore substrate cleavage activities of host protease. Different proteases were diluted into an assay buffer containing the peptide substrate. Only substrate reaction without protease served as a negative control. Assays were conducted in black 96-well plates.

To assess whether these cellular proteases possess similar hydrolytic functions with DENV NS2B/NS3^pro^ and can cleave its substrate, we conducted *in vitro* hydrolysis assays to evaluate the activity of the nine-serine protease activity. Due to challenges in expressing CPD, we focused on expressing the remaining eight proteases and DENV NS2B/NS3^pro^ in *Escherichia coli* (Fig. 3B). For the fluorescence resonance energy transfer (FRET) assay, we employed a peptide substrate Abz-RRRRSAGY-NO_2_, which contains an Abz fluorophore and a NO_2_ quench group. Cleavage of the arginine-serine (R-S) peptide linkage resulted in increased fluorescence signal when excited at 340 nm, exhibiting a wide dynamic range (Fig. 3A). The results revealed that six of the cellular proteases (ABHD2, ACOT2, LYPA2, OTUB1, OVCA2, and PAFAH1B1) exhibited cleavage activities exceeding 60% compared to NS2B/NS3^pro^. On the other hand, RNPEP and SYNGR2 demonstrated only slight activity against the NS2B/NS3^pro^ substrate (Fig. 3C).

### ACOT2 functions at the DENV replication stage

**Fig 4:**
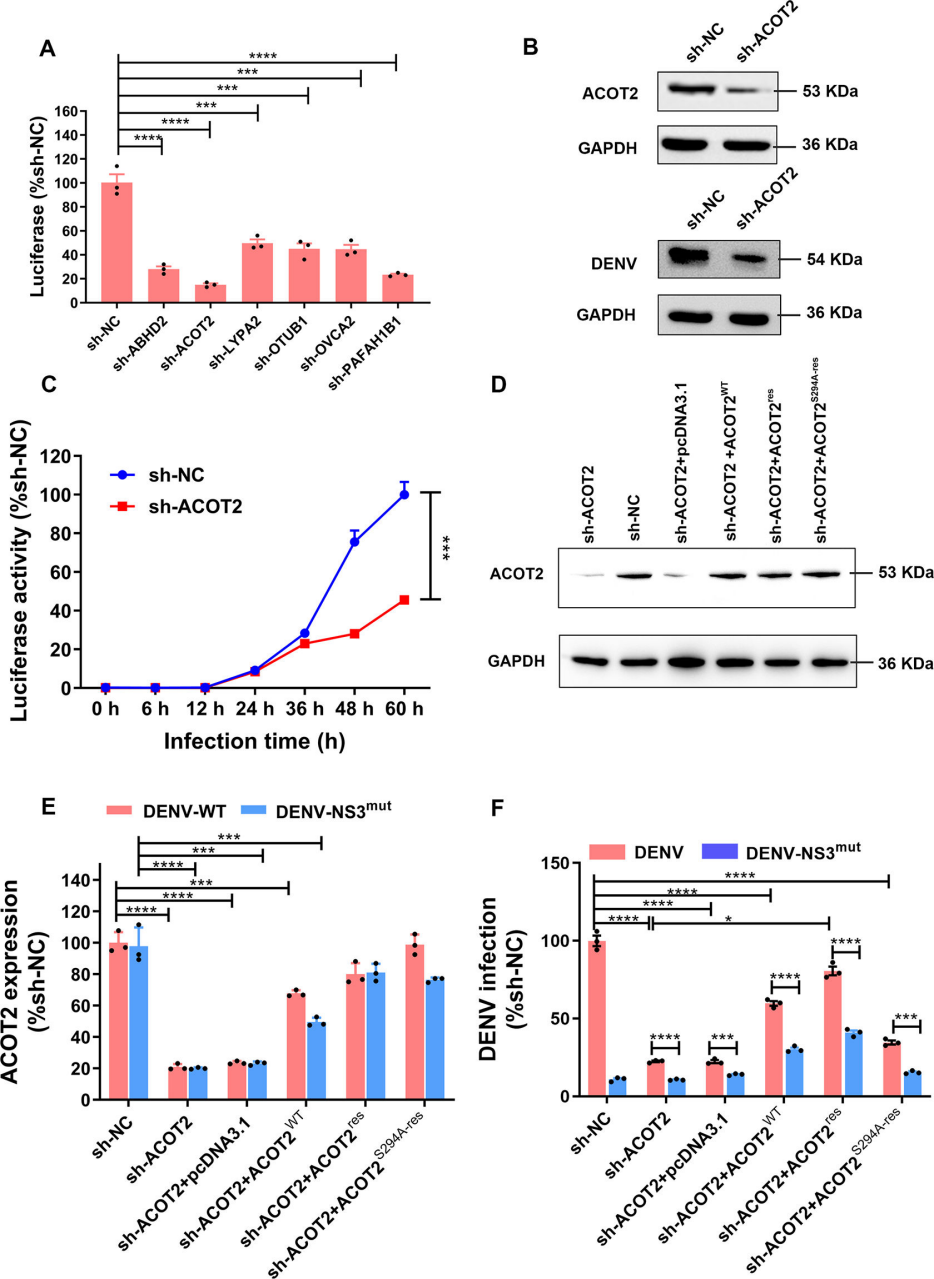
ACOT2 functions at the DENV replication stage. (**A**) The luciferase activity of DENV on different host protease knockdown cell lines, Y-axis represents the percentage of luciferase activity of different knockdown cell lines relative to the A549 cell line (sh-NC) after infection with DENV. (**B**) Western blot detected the expression of endogenous ACOT2 and production of progeny virus in sh-NC and sh-ACOT2 A549 cell line (sh-ACOT2) with infected DENV. Total cell lysates were immunoblotted with anti-ACOT2, anti-DENV Envelope, and anti-GADPH antibodies (loading control). (**C**) The growth curve of DENV in sh-NC and sh-ACOT2. Detected the luciferase activity at 0 h, 6 h, 12 h, 24 h, 36 h, 48 h, and 60 h after DENV-infected, and infection was expressed as a percentage relative to that sh-NC. Graphpad Prim was utilized to plot the growth curves and calculate the statistical significance. (**D**) Detected the expression of ACOT2 in sh-NC, sh-ACOT2, and sh-ACOT2 which overexpressed ACOT2^WT^, ACOT2^res^, and ACOT2^S294A-res^, respectively. (**E**) qRT-PCR examination of the expressions of ACOT2^WT^, ACOT2^res^, and ACOT2^S294A-res^. DENV replicon (DENV) and DENV mutant replicon (DENV-NS3^mut^)-infected sh-NC, sh-ACOT2, and sh-ACOT2 which overexpressed ACOT2^WT^, ACOT2^res^, and ACOT2^S294A-res^, respectively. qRT-PCR then examined ACOT2 in each experimental group, and the expression was shown as a percentage relative to that sh-NC. (**F**) ACOT2 rescued DENV replication. RNA levels of DENV in different experimental groups were detected by qRT-PCR, and DENV infection was then expressed by a percentage relative to that sh-NC. Graphs show mean ± SEM (*n* = 3 biologically independent experiments). *P < 0.05; ***P < 0.005; ****P < 0.001.

We further verify the biological effects of the six-host proteases on DENV proliferation in cell-based assays. To do this, we first designed short hairpin RNAs (shRNAs) and inserted lentiviral vectors to knock down the target gene expressions in A549 cells. We chose the best effect shRNA (Fig. S3) and constructed the knockdown A549 cell lines, respectively. We then infected DENV in different knockdown A549 cell lines, and the results showed that DENV proliferation was reduced by more than 50% compared to the A549 cell line (sh-NC) (Fig. 4A). Among the proteases, ACOT2 exhibited the highest hydrolytic activity and had the most significant impact on virus proliferation. Therefore, we focused on investigating the role of ACOT2 in virus infection. We used western blot (WB) analysis to examine the down-regulation of ACOT2 expression and its effect on virus proliferation in A549 cells. The expression of ACOT2 was significantly decreased, resulting in attenuated production of progeny virus (Fig. 4B).

To determine which stage of the virus life cycle ACOT2 affected, we compared the growth curve of DENV between the sh-ACOT2-A549 cell lines (sh-ACOT2) and A549 cells (sh-NC). The luciferase activity in the sh-ACOT2 remained consistent with the sh-NC before 12 hpi. However, after 24 hpi, the activity in the sh-ACOT2 dramatically decreased (Fig. 4C). This finding indicated that ACOT2 played a crucial role in DENV proliferation during the replication stage rather than the entry stage. To understand how ACOT2 functioned in viral replication, we investigated whether it cleaves virus polyprotein precursors like the DENV- NS3^pro^. We generated wild-type DENV replicon (DENV) and DENV mutant replicon containing the introduction of a single serine to alanine substitution at the Ser135 position of DENV NS3 protein (DENV-NS3^mut^). RNAs of these replicons were transcribed *in vitro* and then transfected into sh-NC and sh-ACOT2. The results showed that the DENV replication efficiency, infected with DENV-NS3^mut^ in sh-NC, was dramatically decreased, and it was also significantly reduced in sh-ACOT2 which was both infected with DENV and DENV-NS3^mut^ (Fig. 4E and F).

Next, we constructed a series of plasmids to investigate the rescue potential of ACOT2. These plasmids included wild-type ACOT2 (ACOT2^WT^), RNA interference-resistant ectopic ACOT2 (ACOT2^res^), and an inactivated ACOT2^res^ with a catalytic residue S294A mutant (ACOT2^S294A-res^). We transfected an equal amount of each plasmid and replicon RNAs of DENV or DENV-NS3^mut^ into sh-NC and sh-ACOT2 to measure their ability to rescue virus replication. At 24 hpi, the ACOT2^WT^ plasmid rescued approximately 70% of the protein expression level compared to the sh-NC control. The ACOT2^res^ and ACOT2^S294A-res^ plasmids performed even better than ACOT2^WT^, achieving a rescue level exceeding 85% (Fig. 4D and E). The great thing was that overexpression of ACOT2^res^ in sh-ACOT2 also rescued the replication activity of both DENV and DENV-NS3^mut^ (Fig. 4F). The RNA levels of DENV and DENV-NS3^mut^ in sh-ACOT2 were rescued to approximately 80% and 40% of those in sh-NC, respectively (Fig. 4F). However, the rescue efficiencies of ACOT2^WT^ and ACOT2^S294A-Res^ were not optimal (Fig. 4F). Taken together, these findings demonstrated that ACOT2 is a critical host factor involved in assisting virus replication during the replication stage of DENV infection.

### ACOT2 hydrolyzes DENV polyproteins to promote virus replication

**Fig 5:**
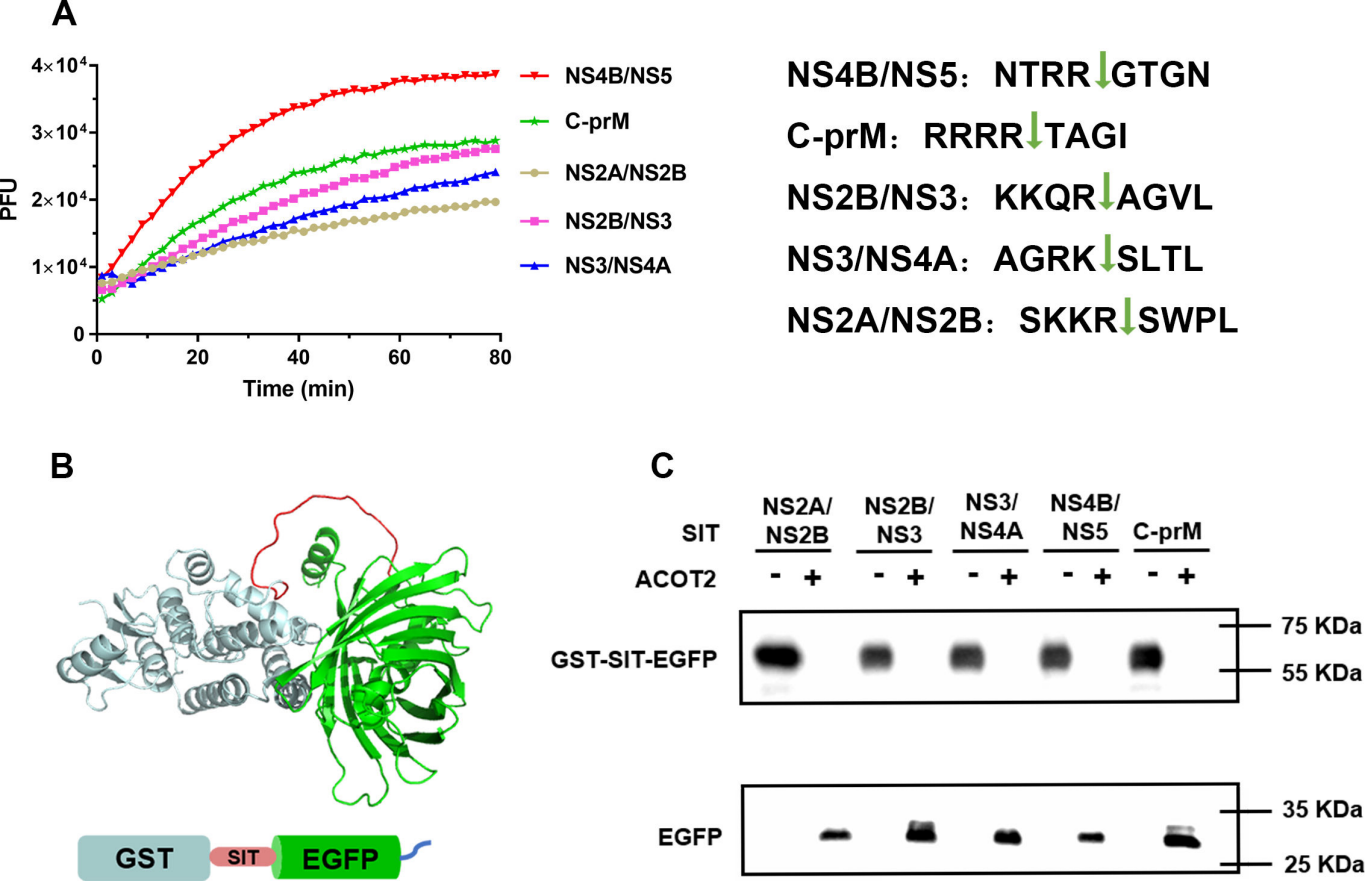
ACOT2 hydrolyzed DENV viral polyproteins. (**A**) ACOT2 cleavaged the polypeptide substrates of NS2B/NS3^pro^. ACOT2 was diluted into a test buffer containing the different polypeptide substrates. Assays were tested in black 96-well plates. (**B**) The structure of recombinant model proteins (GST: aqua; EGFP: green; Polypeptide substrate sequence of NS2B/NS3^pro^ (SIT): rose color; His-tag: blue). (**C**) The hydrolytic activity of ACOT2 on model polyproteins. The reaction products were detected by His-tag which assay western blot. The names and positions of detected SIT and proteins are shown at the up and left, respectively.

ACOT2 has been identified as an acyl-CoA thioesterase that plays a role in the hydrolysis of fatty acyl-CoAs, converting them into free fatty acids and coenzyme A. To investigate the mechanism by which the host factor ACOT2 assisted in DENV replication, we expressed and purified ACOT2 in *E. coli*, and synthesized polypeptide substrates representing cleavage sites within the DENV polyprotein, using the principle of FRET *in vitro*. Our results demonstrated that ACOT2 effectively hydrolyzed all the substrates of NS2B/NS3^pro^, with a preference for cleaving the NS4B-NS5 polypeptide (Fig. 5A).

To further explore the cleavage efficiency of ACOT2, we wish to test whether it could cleavage the polyprotein substrate. However, we encountered difficulties in the expression of viral polyprotein precursors *in vitro*. Thus, we constructed recombinant model proteins that mimic viral polyproteins by inserting each viral polypeptide substrate sequence of NS2B/NS3^pro^ (SIT) between GST and EGFP protein, with a 6 × His-tag on the C-terminus (Fig. 5B). We also constructed the negative control recombinant model protein (NCP) which was inserted the motif recognition by Tobacco Etch Virus protease (TEV) between GST and EGFP protein. The 6 × His-tag western blot detection revealed that ACOT2 effectively cleaved the viral substrate of NS2B/NS3^pro^ within the model proteins, but not in NCP (Fig. 5C; Fig. S5B). Collectively, these findings indicate that the host protease ACOT2 could specifically cleave the viral substrate of NS2B/NS3^pro^ during the replication stage, thereby assisting in the replication of DENV.

### The mechanism of ACOT2 recognizes and hydrolyzes polypeptide substrates

**Fig 6:**
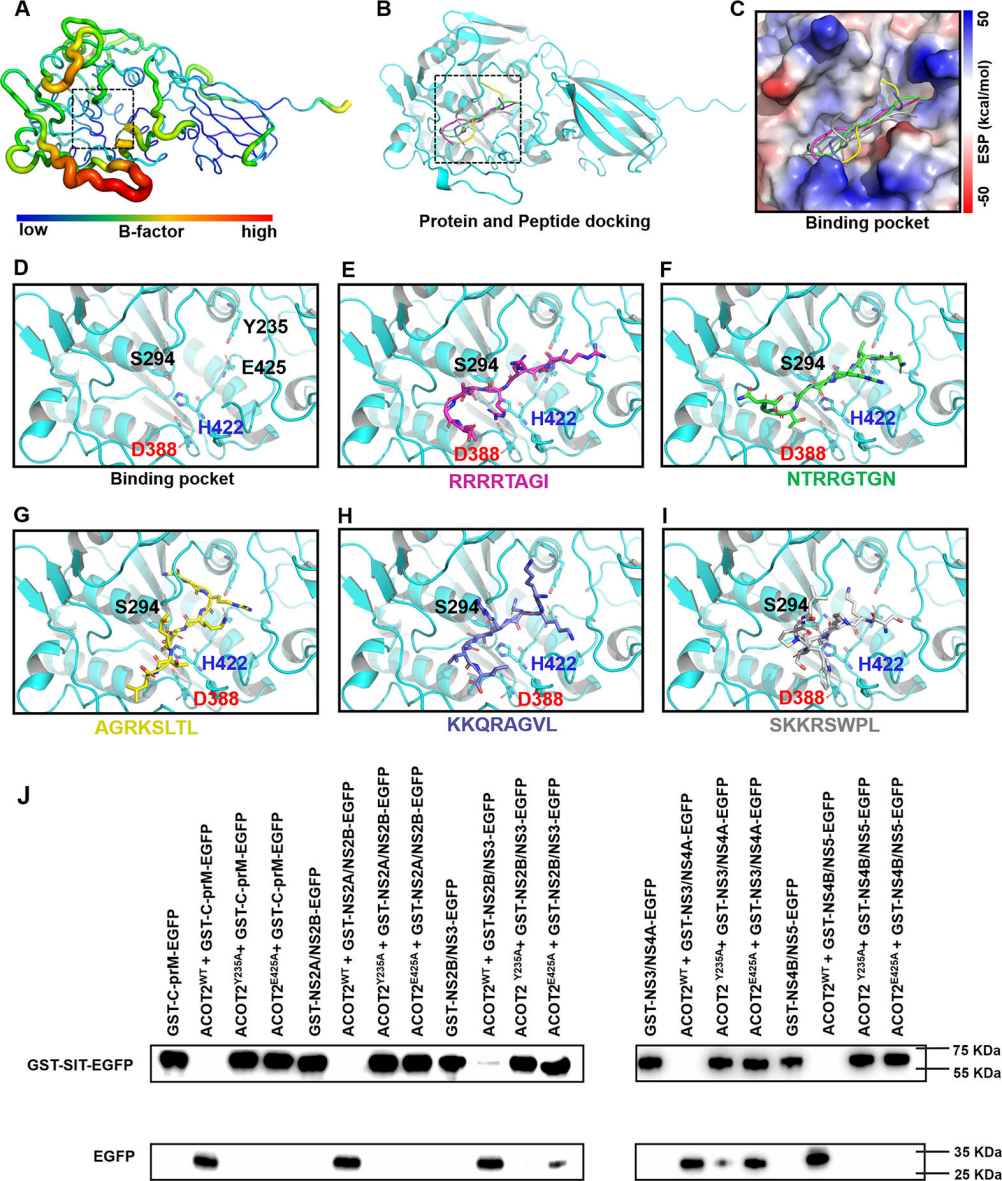
Binding mode between peptides and ACOT2. (**A**) The structure of ACOT2 is shown as a b-factor to indicate that the catalytic center is stable in the structure. (**B**) HPEPDOCK server prediction for five peptide docking results where the receptor is represented in cartoon structure. (**C**) The electrostatic surface potential of the binding pocket. (**D**) The catalytic centers of ACOT2 are shown in the center of the image. The ACOT2 catalytic triad is displayed as sticks and the protease is displayed as cartoon. (**E through I**) The binding poses of the five peptides in the binding pocket, the peptides are shown as sticks of different colors. (**J**) The cleavage activity of ACOT2^WT^; ACOT2^Y235A^ and ACOT2^H425A^ on DENV polypeptides.

Although ACOT2 is capable of hydrolyzing all polyprotein substrates of DENV NS2B/NS3^pro^, the efficiencies vary. To understand the molecular mechanism of ACOT2, docking models were employed to simulate the complex formation between the protease and polypeptides. Unlike protein-ligand docking, where the binding site is typically known, information regarding the binding site for peptides is often unavailable. Moreover, peptides are more flexible and lack a stable conformation prior to binding to a protein. To effectively account for peptide flexibility during docking, we used HPEPDOCK, a program that enables rapid modeling of peptide conformations and global sampling of binding. The docking simulations revealed that the five peptides tended to bind to the substrate active site of the ACOT2, which is a grooved pocket. Figure 6B and C depicts the peptides embedded within this pocket, with the positively charged side of the peptide preferentially binding to the right side of the pocket. Two polar residues (Y235 and E425) are located on the right side of the pocket and their electronegative side chains readily form electrostatic interactions with positively charged residues. This region appears to be important for peptide binding (Fig. 6D through I).

To elucidate the crucial role of Y235 and E425 in substrate binding, we mutated these amino acids to alanine and examined their substrate hydrolytic activity. Western blot results demonstrated that ACOT2^Y235A^ and ACOT2^E425A^ exhibited different hydrolytic activity on DENV polypeptides. Upon mutation of both Y235 and E425, ACOT2 completely lost its protease activity and could no longer cleave the viral substrates of C-prM, NS2A-NS2B, and NS4B-NS5. In comparison, ACOT2^Y235A^ and ACOT2^E425A^ mutations showed slight activity on polypeptides of NS2B-NS3 and NS3-NS4A (Fig. 6J). These findings, along with the FRET assay, demonstrated that ACOT2 preferentially hydrolyzes viral substrates of NS4B-NS5 and C-prM, and Y235 and E425 are essential for the recognition and binding of polyproteins.

**Fig 7:**
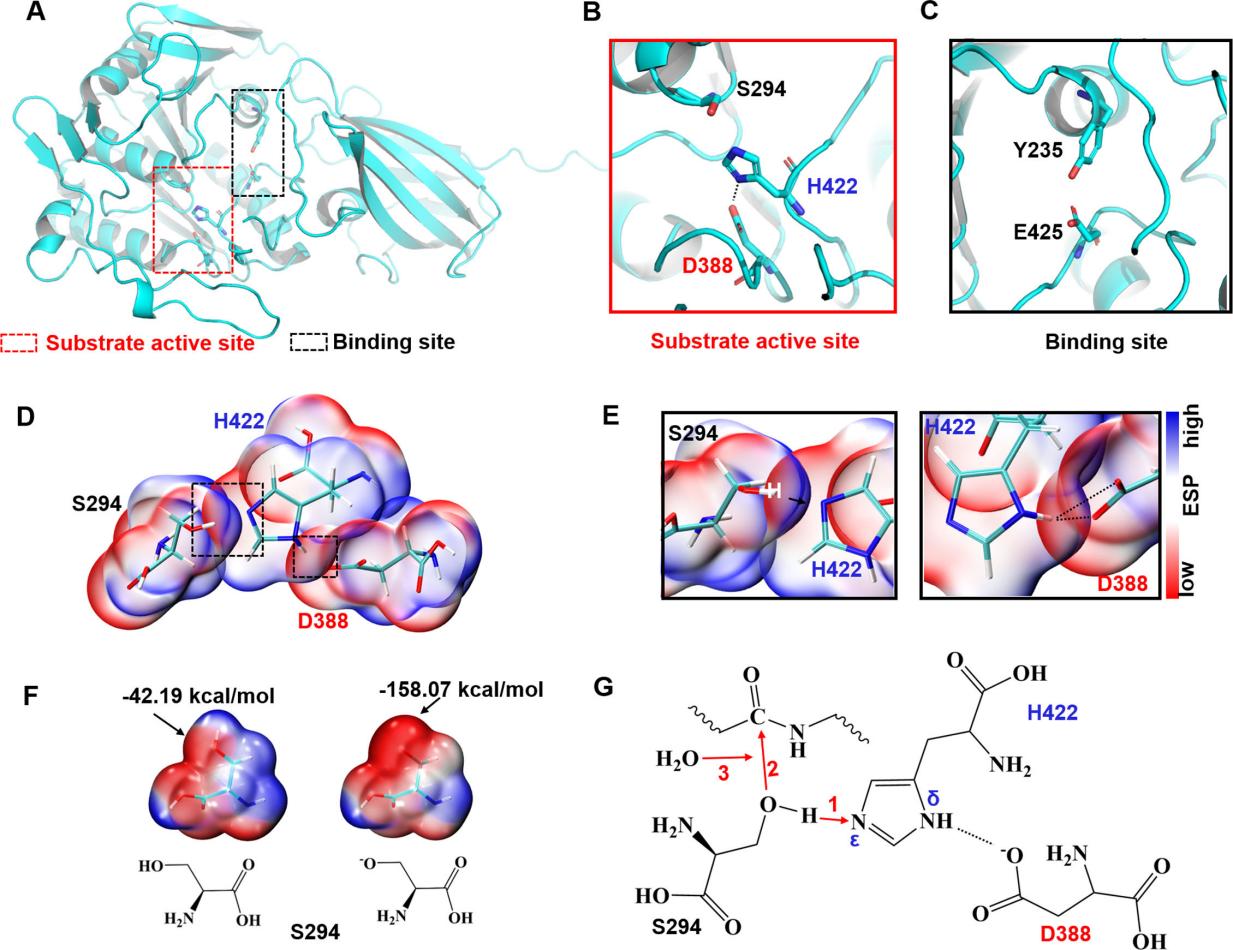
Structural basis of substrate catalysis. (**A-C**) Substrate active site and binding site. (**D-E**) ESP-mapped molecular van der Waals surface of the catalytic triad. (**F**) ESP before and after S294 deprotonation. (**G**) Schematic diagram of the mechanism of ACOT2-catalyzed peptide hydrolysis.

The protein-peptide binding mode described above involves two aspects of information. First, the binding pocket of ACOT2 can be divided into two regions, one responsible for the binding orientation of the peptide and the other one for the hydrolysis of the peptide. Second, the reported substrate active site of ACOT2 may also serve as the site for peptide hydrolysis (Fig. 7A through C). To further understand the possible catalytic mechanism of the catalytic triad, quantum chemical calculations were performed to analyze the electrostatic characteristics of the triad. As depicted in Fig. 6D and E, a hydrogen bond exists between D388 and the amino group at the H422 δ site, stabilizing the conformation of H422 and ensuring that the nitrogen atom at the H422 ε site points toward S294. This alignment assists H422 in capturing the proton from S294. We then calculated the electrostatic surface potential before and after the deprotonation of S294. The potential of the hydroxyl oxygen atom of the serine side chain dropped from −42.19 to −158.07 kcal/mol upon losing the proton (Fig. 7F). Consequently, the S294 hydroxyl oxygen atom can attack the peptide bond, forming an unstable C-O bond with the C atom in the peptide bond. Ultimately, the newly formed C-O bond undergoes hydrolysis, resulting in the complete severing of the peptide (Fig. 7G). While we analyzed the molecular mechanism of ACOT2-catalyzed peptide hydrolysis based on the protein-peptide binding mode and related electrostatic features, further research is warranted to fully understand the microscopic mechanism of ACOT2 binding and peptide hydrolysis.

## DISCUSSION

DENV is a highly prevalent arbovirus worldwide, causing an estimated 390 million infections annually, with approximately 96 million cases resulting in noticeable disease symptoms. Given the significant impact of this arboviral disease on human health, it is crucial to gain a deeper understanding of the viral replication process within host cells to develop effective strategies for disease control. In this study, we employed an FP probe, that directly binds to serine residues to capture serine proteases with altered expression in DENV-infected host cells using comparative ABPP. Through this approach, we identified the host serine protease ACOT2 as a promoter of DENV proliferation by hydrolyzing viral polyproteins. ACOT2 exhibited similar hydrolytic activity to DENV NS2B/NS3^pro^ but displayed different substrate preferences.

It is worth noting that the hydrolysis of DENV polyprotein normally occurs within the cytoplasm, while ACOT2 has been reported to be localized in mitochondria. This raises the question of how ACOT2 participates in the processing of viral polyproteins. To address this, we conducted localization experiments using ACOT2 antibodies. Surprisingly, contrary to previous reports, ACOT2 was found to be abundant in the cytoplasm, except of its localization in mitochondria (Fig. S4). However, we did not find significant interaction between ACOT2 and DENV proteins in the co-immunoprecipitation assay (data not shown). Upon DENV infection of host cells, the virus modifies cellular membranes to establish replication complexes, leading to a significant rearrangement of host cellular membranes and accelerated lipid metabolism (26). Enzymes involved in these pathways, including ACOT2, may exhibit altered activities and expression levels. In fact, several members of the acyl-CoA thioesterases (ACOTs) family have been implicated in intervening in virus infection. For instance, ACOT7 was proved to be upregulated in liver cancer cells infected with HBV (27). ACOT8 not only interacted with HIV nef protein to mediate nef-induced down-regulation of CD4 cell-surface expression but also identified interaction with HSV-1 infected cell protein 0 (ICP0) (28, 29). Recently, a study reported the phenomenon that ACOT2 significantly potentiated DENV2 protein translation, genome replication, and infectious virus release (30). However, the complex interplay between ACOT2 and DENV is still not completely understood, and the regulatory machinery of ACOT2 remains intriguing. Moreover, an in-depth investigation into the function of ACOT2 in animal models of DENV infection is urgently warranted. To date, plenty of studies have confirmed that the lipid landscape of host cells is significantly altered during DENV infection to assemble membrane platforms and support viral replication. ACOTs are crucial elements in the biosynthesis of complex lipids that form these membranes (31, 32). There are 10 identified human ACOT enzymes, which are distributed in cytoplasm, mitochondria, and peroxisomes (33). Whether the other ACOTs participate in DENV infection is needed to fully understood. Nevertheless, the multiple biological functions of differential distribution of ACOTs in other organelles within virus proliferation deserve further investigation.

In our study, we unveil the unique function of ACOT2 as a protease and identify its polypeptide substrates for the first time. Furthermore, we deduce the binding pocket of these substrates and identify Y235 and E425 as critical residues for substrate binding and recognition. Quantum chemical calculations provide further insights into the molecular mechanism by which ACOT2 catalyzes peptide hydrolysis. Most importantly, these findings not only uncover a novel function of ACOT2 but also expand our understanding of its biological significance in viral replication.

## MATERIALS AND METHODS

### Cell lines and virus

A549, Baby Hamster Syrian Kidney (BHK21), and Human Embryonic Kidney (HEK-293T) cell lines utilized in the experiment were obtained from ATCC. The cells were maintained in Dulbecco’s modified Eagle’s medium (DMEM) (Gibco), supplemented with 10% fetal bovine serum (FBS) (Gibco), 100 U/mL penicillin, and 100 mg/mL streptomycin (Gibco), at 37°C with 5% CO_2_ in a humidified incubator.

The plasmids of the full-length genome of Dengue virus type 2 strain TSV01(DENV-2) and DENV-2 replicon (GenBank: AY037116.1) fused with Renilla luciferase were generously provided by Bo Zhang from Wuhan Institute of Virology. The DENV-2 plasmid was linearized and transcribed to RNA *in vitro*. The resulting RNAs were transfected into BHK21 cells to generate the DENV luciferase virus.

### Virus titration

Plaque assays for DENV-2 luciferase virus were performed as previously described (34). BHK21 cells (2 × 10^5^ per well) were seeded in a 24-well plate (Nunc, Denmark) and cultured for 24 hours. Viral supernatant was diluted at 10-fold and loaded (0.2 mL) onto each well of BHK21 cells. After incubating for 1 hour at 37°C, the virus inoculums were replaced with 0.5 mL of 0.8% methylcellulose Aquacide (Sigma-Aldrich) containing 2% FBS in DMEM. After 4 days of incubation at 37°C, the cells were stained with 1% of crystal violet, followed by visual counting of plaques. Then, A549 cells were infected at MOI of 1, 2, 3, 5, and 10 and luciferase activities were tested at 24, 48, 72, and 96 hours, respectively. The virus replication reached the highest level after 72 hours at MOI of 5.

### Optimization of FP labeling concentration and time

To achieve the best labeling efficiency, we optimized the labeling concentration and time of the FP probe. First, the FP probe was synthesized according to the previous method (35). Then, A549 cells were infected with DENV for 72 hours and the cell lysates were reacted with FP probes at various concentrations. The TAMRA-azide reagents were incubated for 30 minutes and detected by in-gel fluorescence. At concentrations above 50 µM, the labeling efficiency showed no significant difference. Followingly, we optimized the labeling time of FP at 50 µM for different times using the same protocol. FP with strong signals at 30 minutes was chosen as the final reaction condition.

### Proteome extraction and labeling

A549 cells were infected with DENV-2 luciferase virus at MOI of 5 and incubated for 72 hours. The cells were then harvested by centrifugation and washed twice with chilled phosphate-buffered saline (PBS). The collected cells were resuspended in PBS containing 0.1% NP-40, homogenized using sonication, and centrifuged to separate the soluble fraction. The protein concentrations were determined using the bicinchoninic acid (BCA) assay and diluted to a concentration of 2 mg/mL in PBS.

The proteome samples were mixed with FP and incubated at 25°C for 1 hour. Click reactions were performed using 200 µM TAMRA-azide or biotin-azide, 1 mM Tris (2-carboxyethyl) phosphine hydrochloride (TCEP), 100 µM Tris [(1-benzyl-1H-1,2,3-triazol-4-yl) methyl] amine (TBTA), and 1 mM CuSO_4_ at 25°C for 30 minutes. The samples labeled with FP and TAMRA-azide were analyzed using SDS-PAGE and imaged on an Azure Biosystems instrument (Azure C400, USA). The samples labeled with biotin-azide samples were incubated with streptavidin agarose resins (Thermo Fisher Scientific, USA) for 3 hours, followed by centrifugation and washing with 0.2% SDS in PBS (5 mL for 10 minutes), PBS (3 times with 5 mL each), and water (3 times with 5 mL each). The samples were then boiled, fractionated using SDS-PAGE, and silver stained.

### Protein identification and label-free quantification

Samples, both infected and uninfected with DENV-2, were silver stained and cut into four slices for in-gel trypsin digestion. For LC-MS/MS analysis, the resulting digested peptides were separated using a Thermo-Dionex Ultimate 3000 high-performance liquid chromatography (HPLC) system with a flow rate of 0.30 µL/min and a 120-minute gradient elution. The HPLC system was directly connected to a Thermo Scientific Q Exactive mass spectrometer.

A homemade fused silica capillary column [75 µm internal diameter (ID), 150 mm length; Upchurch, Oak Harbor, WA) packed with C18 resin (300 Å, 5 µm; Varian, Lexington, MA) was used as the analytical column. The mobile phase A consisted of 0.1% formic acid, with mobile phase B containing 80% acetonitrile and 0.1% formic acid.

The Q Exactive mass spectrometer was operated in the data-dependent acquisition mode using Xcalibur 2.1.2 software. The analysis began with a single full-scan mass spectrum in the orbitrap, covering a range of m/z 300 to 1,800 m/z at a resolution of 70,000. This was followed by 20 data-dependent MS/MS scans with a normalized collision energy of 27% [high-energy collisional dissociation (HCD)].

Protein identification and label-free quantification were performed using MaxQuant_1.5.5.1. The searches for protein identification were conducted against the UniProt database. The target proteins were identified through comparative ABPP.

### Expression and purification of proteases

The genes encoding the target proteins and DENV NS2B/NS3^pro^ were amplified and subcloned into pGEX-6p-1 vectors. Otherwise, we constructed a model protein, which inserted the polyproteins substrate sequence of NS2B/NS3^pro^ (SIT) into GST and EGFP, with a 6 × His-tag on the C-terminus, and subcloned the model protein into pET-28a vectors. The recombinant plasmids were then transformed into *E. coli* BL21 (DE3) cells. Cells were cultured in LB medium at 37°C until reaching an optical density at OD_600_ of 0.8, and then induced with 0.5 mM isopropyl-D-1-thiogalactopyranoside (IPTG) for 16 hours at 16°C. Subsequently, the cultures were harvested and subjected to sonication in a lysis buffer to homogenize the cells. The resulting lysates were clarified by centrifugation at 18,000 rpm for 30 minutes to remove cellular debris. The supernatants were then applied to a Glutathione-Sepharose resin (GE Healthcare, USA), allowing the nonspecific contaminants to be washed away with the lysis buffer. The target proteins were separated from the GST tag using PreScission Protease, were subsequently eluted from the resin, and further purified using the Superdex 200 column (GE Healthcare, USA).

### Proteolytic activity of proteases

*In vitro* protease activity assays were performed using purified recombinant proteases and fluorescence substrates: Abz-RRRRTAGI-NO_2_ (C-prM), Abz-NTRRGTGN-NO_2_ (NS4B/NS5), Abz-AGRKSLTL-NO_2_ (NS3/NS4A), Abz-KKQRAGVL-NO_2_ (NS2B/NS3), and Abz-SKKRSWPL (NS2A/NS2B), respectively. Briefly, proteases (1 µM) and substrate (20 µM) were incubated in a solution containing 20 mM HEPES, 2 mM DTT, and pH 7.2. The reaction mixtures were then analyzed using Varioskan Flash, and the relative fluorescence units were measured at an excitation wavelength (λex) of 320 nm and an emission wavelength (λem) of 420 nm. Measurements were taken at 2-minute intervals for a duration of 60 minutes. We used ACOT2 to digest the model protein *in vitro*. ACOT2 (1.2 µM) and model proteins (1 µM) were incubated in the buffer containing 20 mM HEPES, 2 mM DTT, pH 7.2 at 30°C overnight. Then the His-tag was detected by western blot to reflect the digestion efficiency of ACOT2.

### Generation of stable knockdown cell lines

The shRNAs were used to downregulate specific genes in cell lines (refer to Table S2 for details). The shRNAs were incorporated into pLenti plasmids, along with two packaging plasmid vectors, pMD2.G and psPAX2. These plasmids were co-transfected into HEK-293T cells and cultured at 37°C for 48 hours. The supernatants were collected and added to A549 cells, which were then incubated for an additional 24 hours. Puromycin was utilized to screen stable knockdown cell lines. The efficiencies of knockdown were assessed using qRT-PCR.

### Real-time quantitative PCR

Total intracellular RNAs were extracted using the EasyPure RNA kit (Transgen Biotech, China) and reverse transcribed into cDNA. The qRT-PCR assays were conducted using the SYBR Premix Green RT-PCR kit (Bio-Rad, USA) according to the manufacturer’s instructions. The amplifications were carried out using a thermocycler (Roche, Switzerland). The primer sequences can be found in Table S3. The transcript levels were determined using the *ΔΔCT* method.

### Virus rescue

The mutations of ACOT2 and DENV replicon were introduced using a site-directed mutagenesis kit (TransGen, China). The sh-ACOT2 cells were transfected with different constructs, including pcDNA3.1, wild-type ACOT2 (ACOT2^WT^), RNA interference-resistant ectopic ACOT2 (ACOT2^Res^), and ACOT2^Res^ with S294A mutate (ACOT2^S294A-Res^). Simultaneously, cells were transfected with a wild-type DENV-2 replicon and a mutate replicon carrying the S135A mutation on NS3. The expression levels of ACOT2 were determined using western blotting and qRT-PCR. The replications of DENV were assessed using the Ranilla luciferase Kit (Promega, USA).

### Indirect immunofluorescence assay

A549 cells were washed with PBS thrice and then fixed with 4% paraformaldehyde at 4°C for 1 hour. Subsequently, the cells were treated with PBST (0.5% TritonX-100 in PBS) at 4°C for 10 minutes. For the detection of endogenous and overexpressed ACOT2, the cells were incubated with a rabbit polyclonal antibody against ACOT2 (Invitrogen, PA5-101403), followed by an incubation with a goat anti-rabbit IgG secondary antibody conjugated with Alexa Fluor 546 (Invitrogen). Mitochondria were visualized using a green fluorescent probe Mito Scene Green I (Everbright USA Inc, M4063S). Nuclei were stained with DAPI (15 µg/ml) at room temperature for 20 minutes. The location of ACOT2 was detected using Super-resolution confocal microscopy (Nikon).

### Computational details

The crystal structure of human mitochondrial acyl-CoA thioesterase (ACOT2 PDB: 3hlk) was used as the docking receptor file (21). HPEPDOCK was used for rapid modeling of peptide conformations and peptide-protein docking (36). HPEPDOCK incorporated peptide flexibility by generating an ensemble of peptide conformations using the MODPEP program and performed protein-peptide docking through a hierarchical algorithm. The docking energy was assessed, and the structure with the lowest docking energy was selected for further analysis of the peptide and ACOT2 binding mode. The necessary theoretical calculations were conducted using Gaussian 03 (37) and Multiwfn (38). The electrostatic surface potential data were generated using the B3LYP/6–31G** level algorithm. Visualization and analysis of model features were carried out using VMD (39) and Open-Source Pymol (https://pymol.org).

## SUPPLEMENTAL MATERIAL

Figure S6C and D msystems.00973-23-s0001.tif ^31^P and ^19^F NMR spectra.

Figure S1 msystems.00973-23-s0002.tif Synthesis and mechanism of FP.

Figure S2 msystems.00973-23-s0003.tif Optimization of FP labeling concentration and time.

Figure S3 msystems.00973-23-s0004.tif Preparation of knockdown cell lines using shRNAs.

Figure S4 msystems.00973-23-s0005.tif The location of cellular ACOT2.

Figure S5 msystems.00973-23-s0006.tif The expression of the model proteins.

Figure S6A and B msystems.00973-23-s0007.tif ^1^H and ^13^C NMR spectra.

Legends msystems.00973-23-s0008.pdf Supplemental figure legends.

Supplemental Tables msystems.00973-23-s0009.pdf Tables S1-S3.

ASM does not own the copyrights to Supplemental Material that may be linked to, or accessed through, an article. The authors have granted ASM a non-exclusive, world-wide license to publish the Supplemental Material files. Please contact the corresponding author directly for reuse.
