# Supplementary figures and images for: Host serine protease ACOT2 assists DENV proliferation by hydrolyzing viral polyproteins
Source: mSystems. 2023 Dec 19;9(1):e00973-23. doi: 10.1128/msystems.00973-23 (PMC10804956; doi:10.1128/msystems.00973-23)

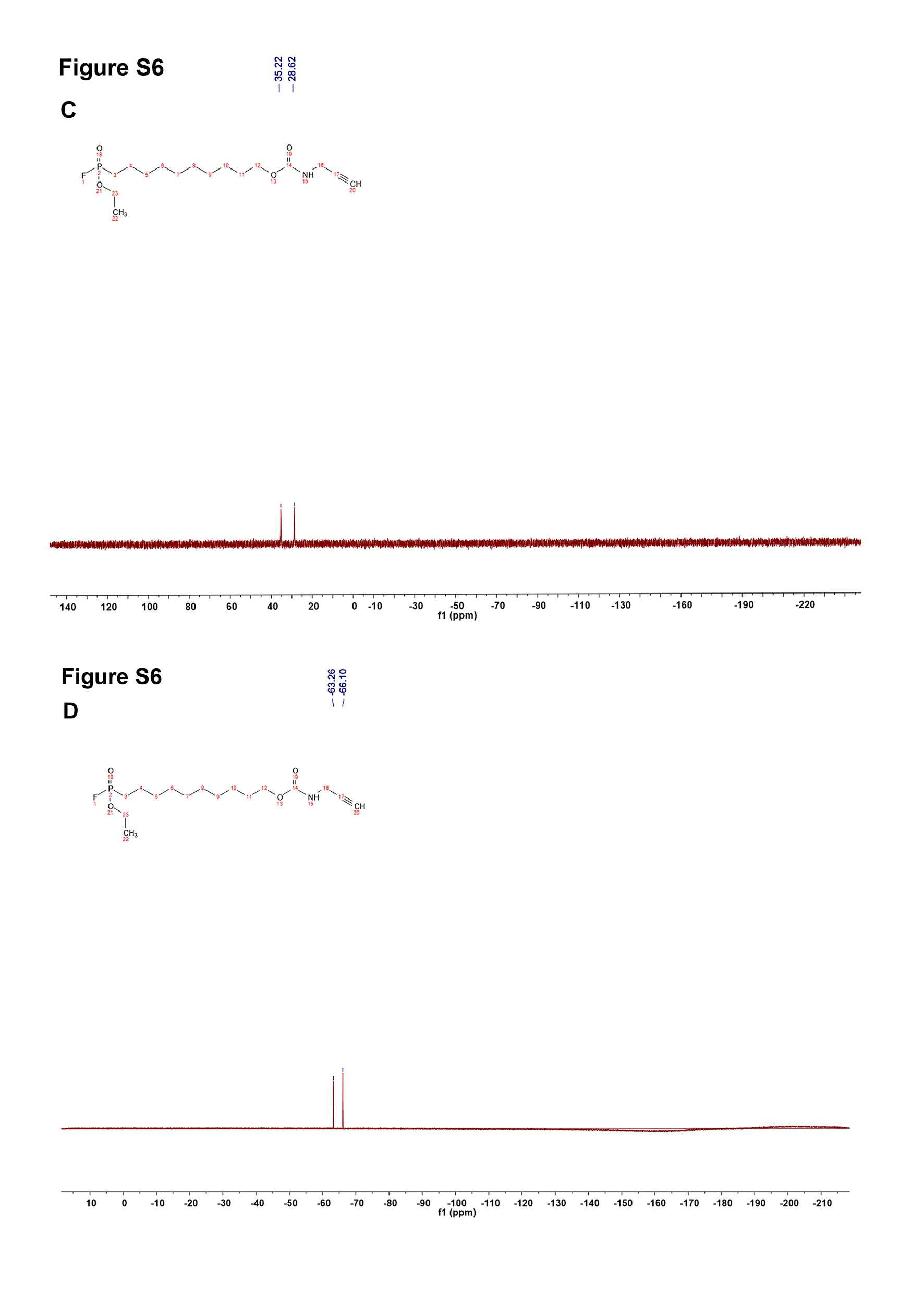

Supplement: Figure S6C and D — 31P and 19F NMR spectra. [file msystems.00973-23-s0001.tif]

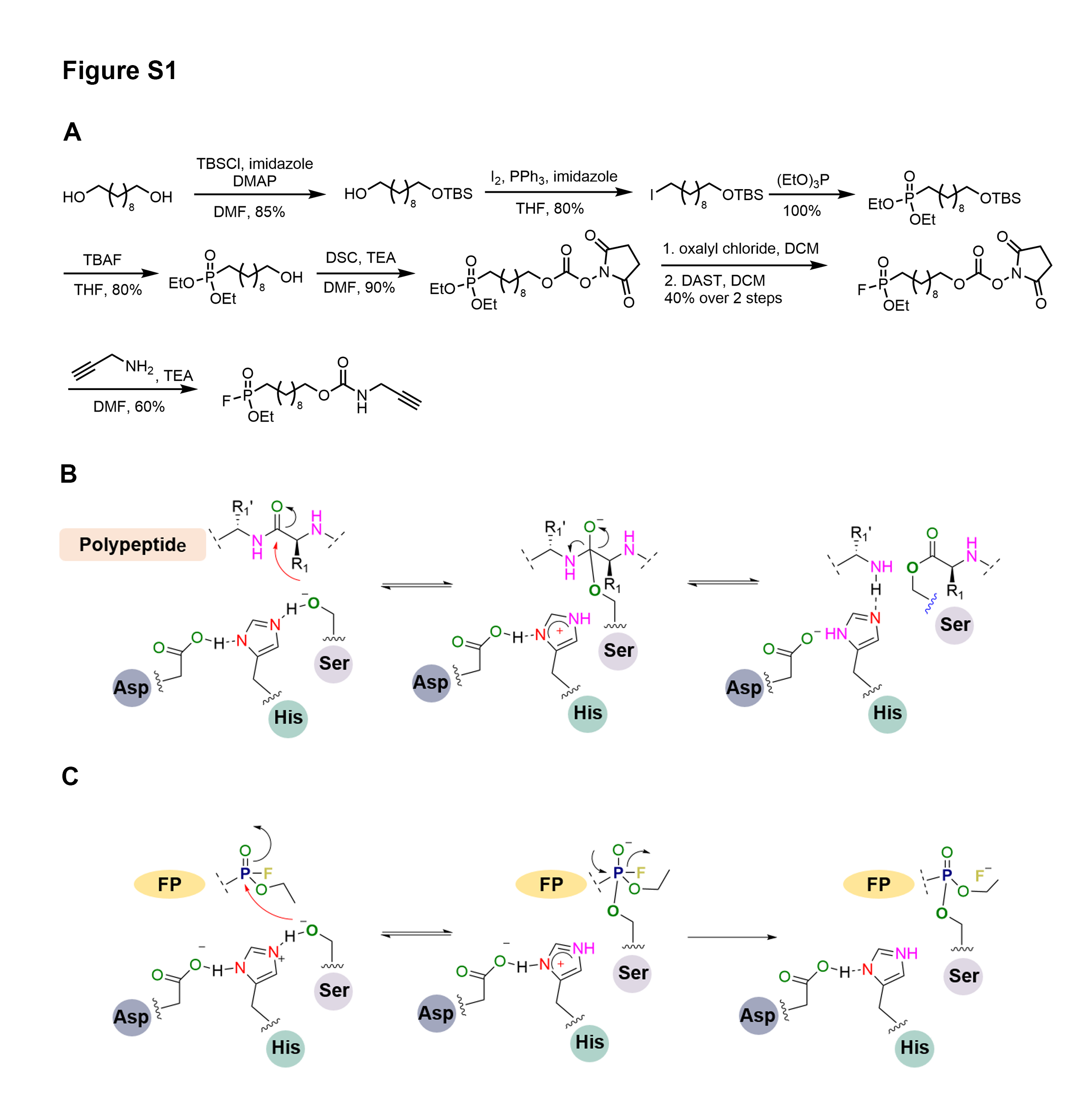

Supplement: Figure S1 — Synthesis and mechanism of FP. [file msystems.00973-23-s0002.tif]

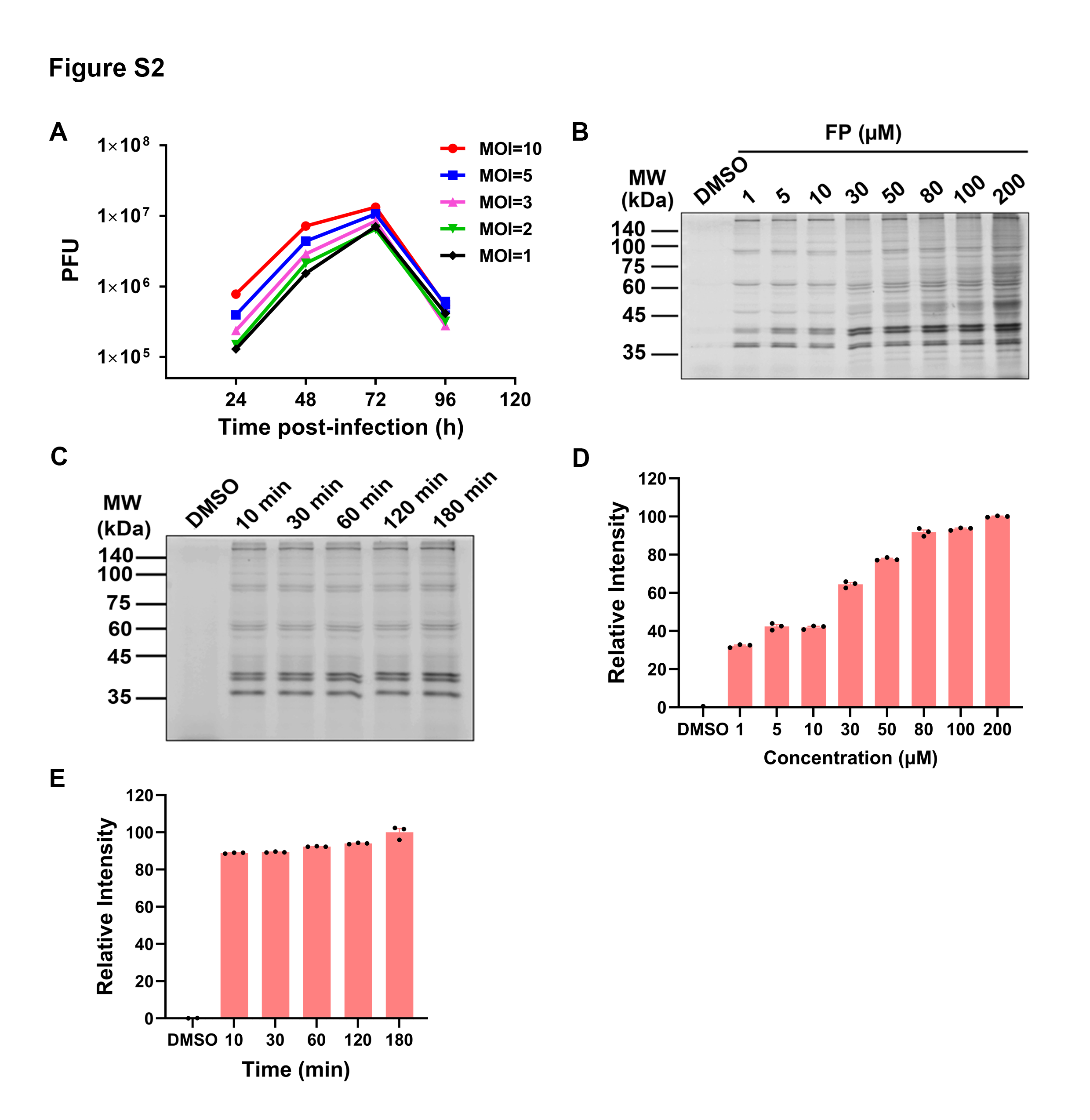

Supplement: Figure S2 — Optimization of FP labeling concentration and time. [file msystems.00973-23-s0003.tif]

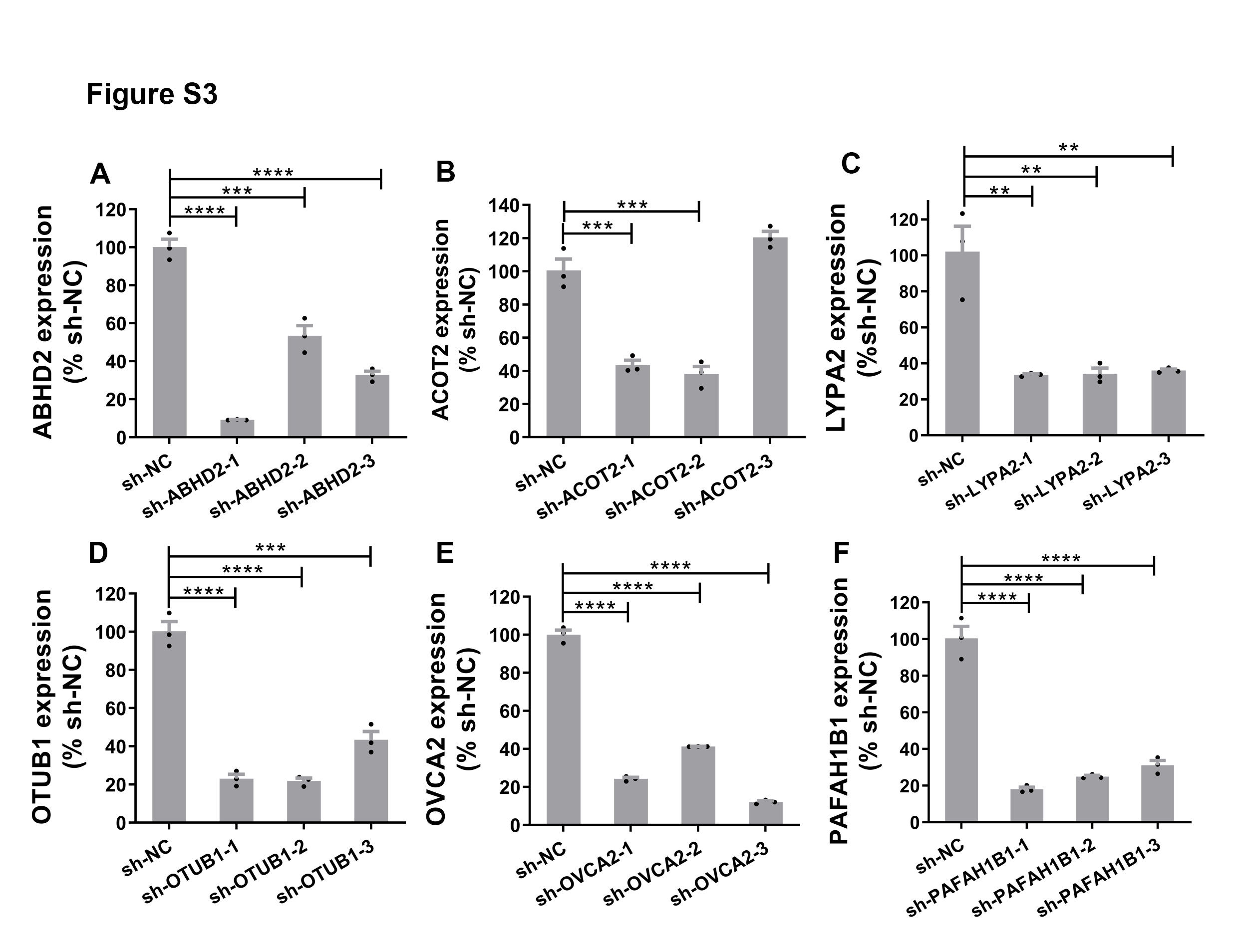

Supplement: Figure S3 — Preparation of knockdown cell lines using shRNAs. [file msystems.00973-23-s0004.tif]

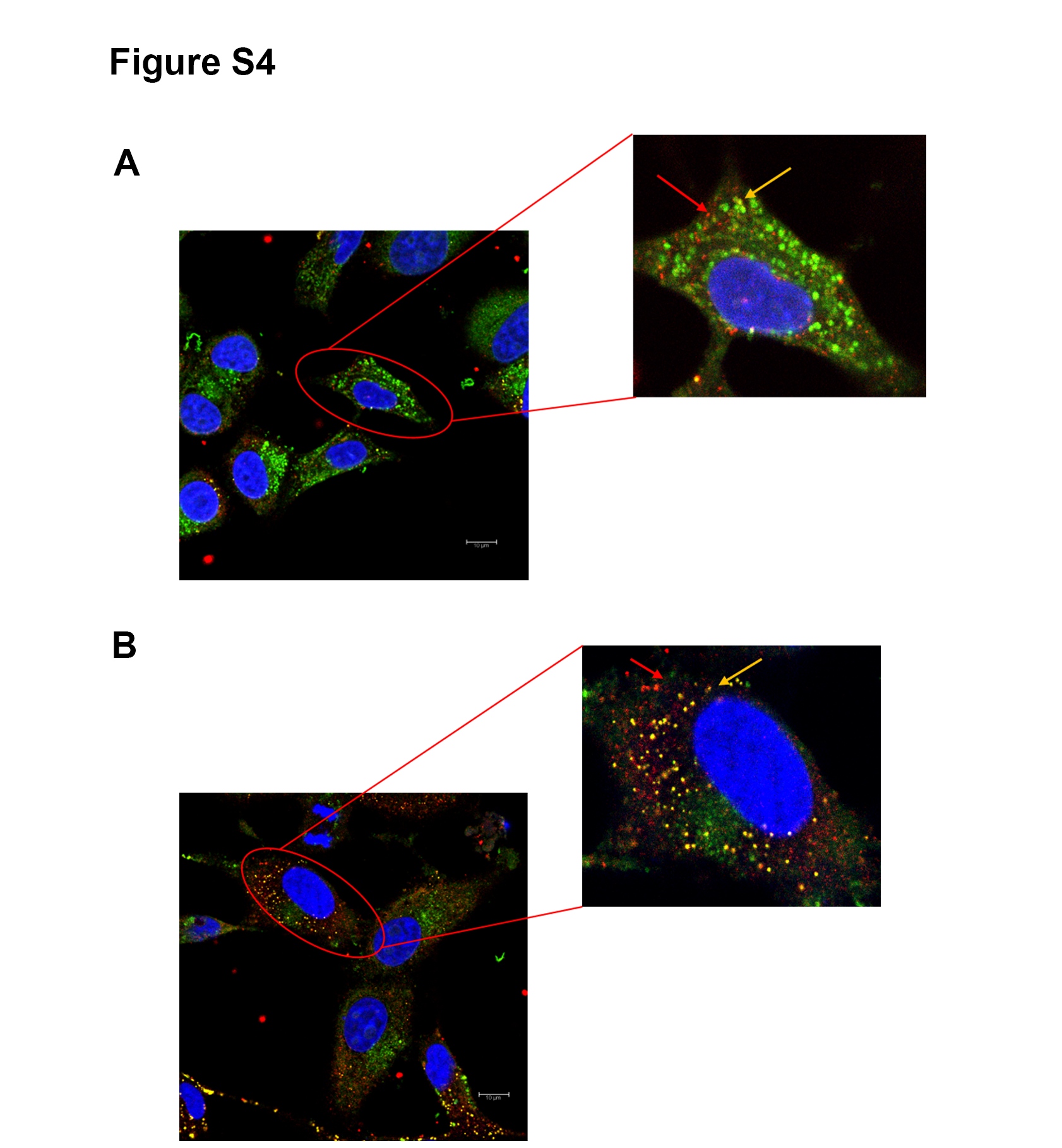

Supplement: Figure S4 — The location of cellular ACOT2. [file msystems.00973-23-s0005.tif]

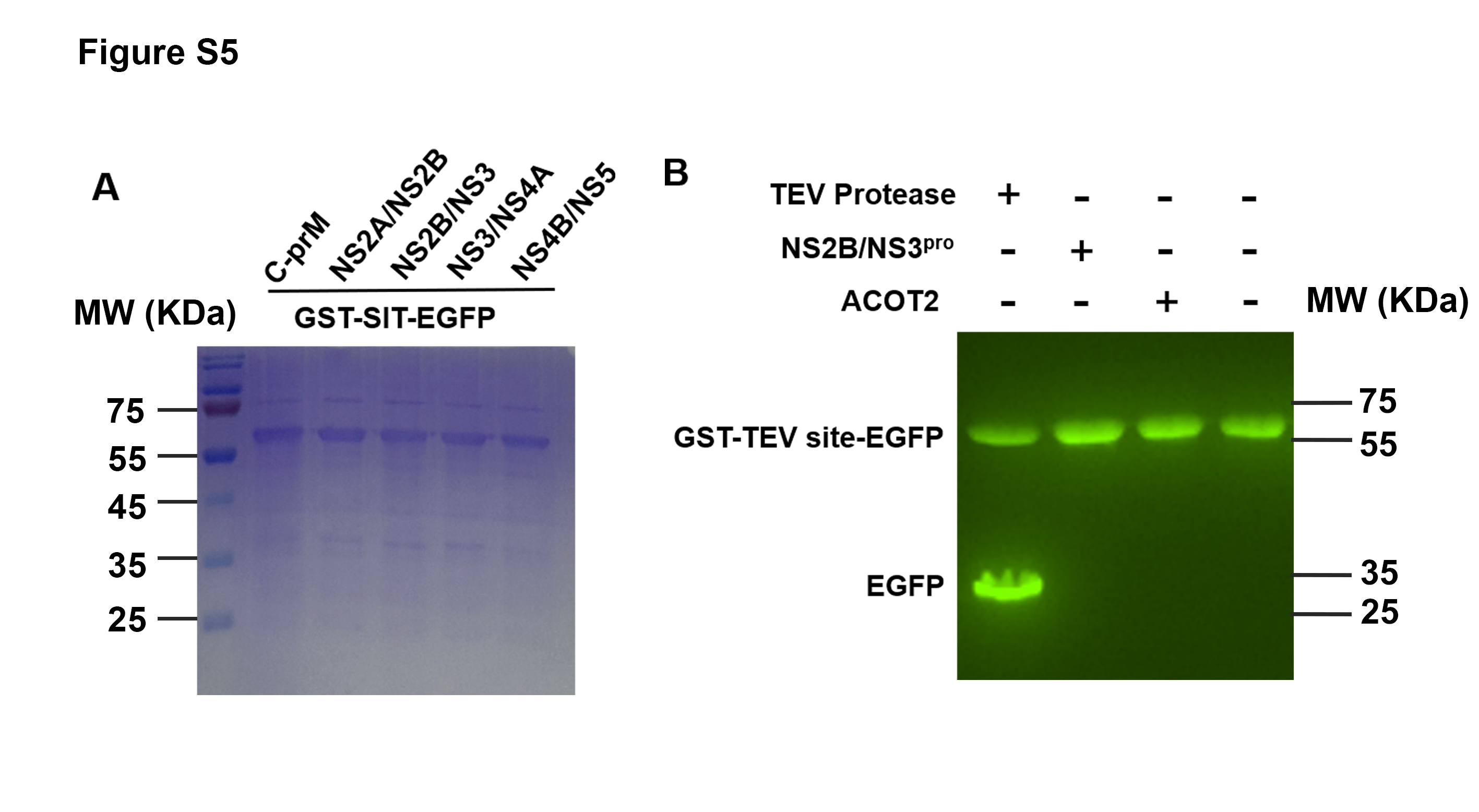

Supplement: Figure S5 — The expression of the model proteins. [file msystems.00973-23-s0006.tif]

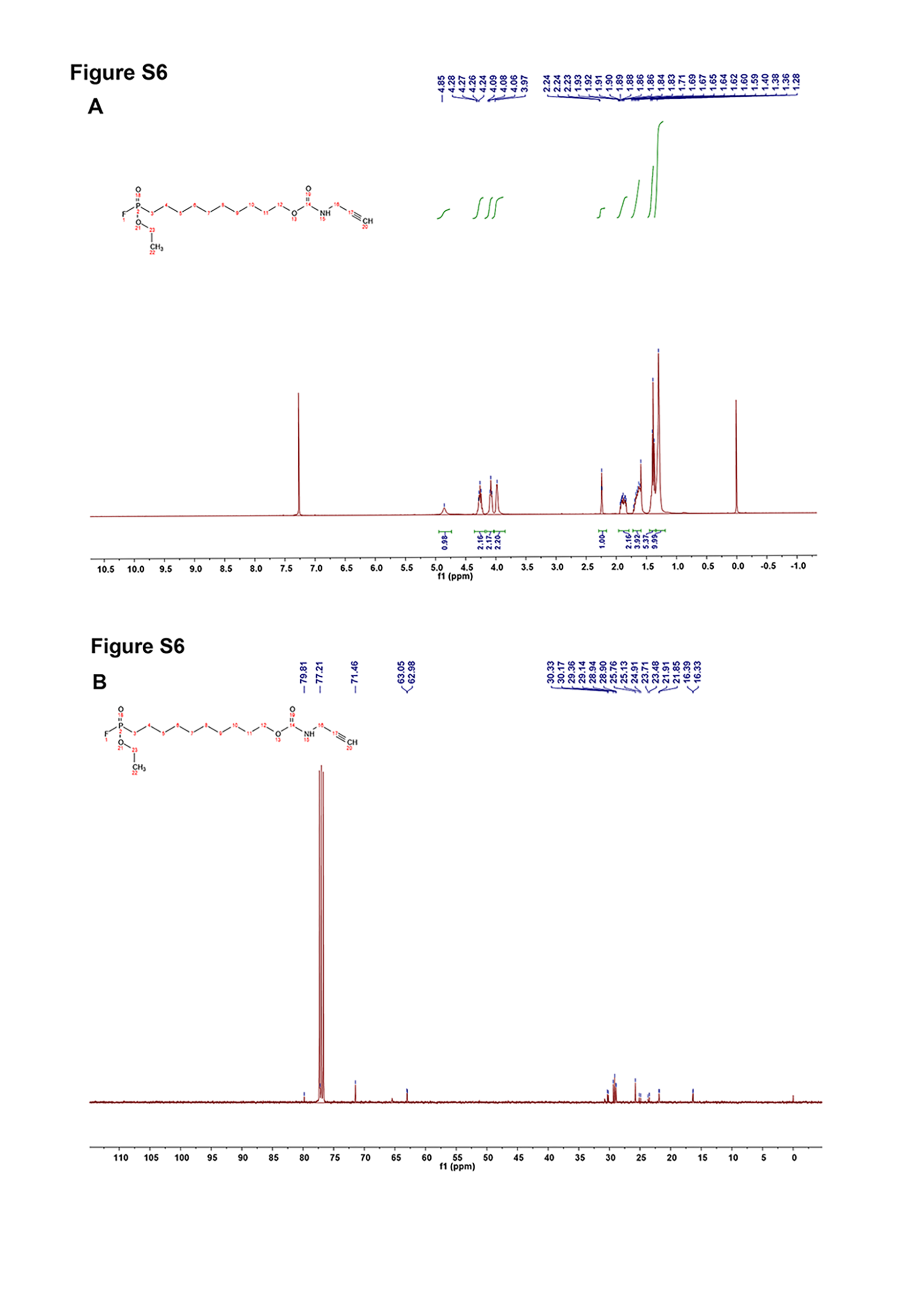

Supplement: Figure S6A and B — 1H and 13C NMR spectra. [file msystems.00973-23-s0007.tif]
