# Supplementary material for: Host serine protease ACOT2 assists DENV proliferation by hydrolyzing viral polyproteins
Source: mSystems. 2023 Dec 19;9(1):e00973-23. doi: 10.1128/msystems.00973-23 (PMC10804956; doi:10.1128/msystems.00973-23)
Supplement: Legends — Supplemental figure legends. [file msystems.00973-23-s0008.pdf]

Supplementary Figure legends

Figure S1. Synthesis and mechanism of FP. (**A**) Synthesis of FP probe. (**B**) The mechanism of serine protease hydrolyzes polypeptide. Serine protease hydrolyzes the ester and amide bonds of protein substrates by engaging the esters and amides at sp2 carbon via a base-activated serine nucleophile. Then, the acyl-protease intermediate is cleaved by a water molecule and the protease is restored to its active state. (**C**) FP probe reacts with serine protease. FP probe utilizes serine protease active site reaction characteristics to covalently react with highly nucleophilic serine-derived oxygen atoms to target proteins active centers.

Figure S2. Optimization of FP labeling concentration and time. (**A**) A549 cells were infected with DENV luciferase in different MOI and infection times. (**B**) Quantification of the intensity of FP concentration-dependent labeling. (**C**) Quantification of the intensity of time-dependent labeling. (**D**) The relative intensity of different FP concentration-dependent labeling. (**E**) The relative intensity of different time-dependent labeling.

Figure S3. Preparation of knock-down cell lines using shRNAs. Each cell line was knocked down using three different shRNAs and compared their efficiency. The knocked down efficiency of the host protein was expressed as a percentage relative to that sh-NC. Graphs show mean ± SEM. (n = 3 biologically independent experiments).

Figure S4. The location of cellular ACOT2. (**A**) The location of endogenous ACOT2. (**B**) The location of overexpressed ACOT2. The cell nuclei were stained with DAPI (blue), ACOT2 was visualized by Alexa FluorTM 546 (red), and the mitochondria were

stained with Mito SceneTM Green I (green).

Figure S5. The expression of the model proteins. (A) The expression of the recombinant model proteins which inserted polypeptide substrate sequence of NS2B/NS3pro (SIT) between GST and EGFP protein. (B) The hydrolytic activity of ACOT2 on negative control recombinant model protein (NCP). NCP, which inserted motif recognition by Tobacco Etch Virus protease (TEV), was digested by TEV, $NS2B/NS3^{pro}$, and ACOT2. The reaction products were analyzed by fluorescence of EGFP.

Figure S6. NMR spectra of FP probes. **(A) ^{1}H NMR** (400 MHz, $CDCl_3$) δ 4.85 (s, 1H), 4.26 (dd, J = 12.1, 5.1 Hz, 2H), 4.08 (t, J = 6.3 Hz, 2H), 3.97 (s, 2H), 2.24 (t, J = 2.3 Hz, 1H), 1.96 – 1.79 (m, 2H), 1.72 – 1.59 (m, 4H), 1.38 (t, J = 7.1 Hz, 5H), 1.28 (s, 10H). **(B) ^{13}C NMR** (101 MHz, $CDCl_3$) δ 79.81, 77.21, 71.46, 63.05, 62.98, 30.33, 30.17, 29.36, 29.14, 28.94, 28.90, 25.76, 25.13, 24.91, 23.71, 23.48, 21.91, 21.85, 16.39, 16.33. **(C) ^{31}P NMR** (162 MHz, $CDCl_3$) δ 31.92 (d, J = 1069.4 Hz). **(D) ^{19}F NMR** (376 MHz, $CDCl_3$) δ -64.68 (d, J = 1069.5 Hz).
