# Supplementary material for: Host serine protease ACOT2 assists DENV proliferation by hydrolyzing viral polyproteins
Source: mSystems. 2023 Dec 19;9(1):e00973-23. doi: 10.1128/msystems.00973-23 (PMC10804956; doi:10.1128/msystems.00973-23)
Supplement: Supplemental Tables — Tables S1-S3. [file msystems.00973-23-s0009.pdf]

Table 1. Serine proteases identified by comparative ABPP

UniProt ID	Protein name	Gene name	MW (kDa)[a]	Ratio of comparative ABPP[b]
P08910	Monoacylglycerol Lipase ABHD2	ABHD2	48	2.54
P49753	Acyl-Coenzyme A Thioesterase 2	ACOT2	53	3.43
O75976	Carboxypeptidase D	CPD	153	2.89
O95372	Lysophospholipase 2	LYPA2	25	1.57
Q96FW1	Ubiquitin Thioesterase OTUB1	OTUB1	31	1.97
Q8WZ82	Esterase OVCA2	OVCA2	24	1.62
P43034	Platelet-Activating Factor Acetylhydrolase 1B Subunit	PAFAH1B1	47	1.60
C9JMZ3	Aminopeptidase B	RNPEP	39	5.42
O43760	Synaptogyrin-2	SYNGR2	25	2.34

[a]MW: Molecular Weight.
[b]The reported ratios are the means from three biological replicates.

Table 2. Sequences of shRNAs

Target	Sequence
NC	5′-TTCTCCGAACGTGTCACGT-3′
ABHD2-1	5′-GAAGCAATACATCCGCACTTT-3′
ABHD2-2	5′-CATTTGCCAATGGGAGCGTAA-3′
ABHD2-3	5′-CGGTACCTGCACAGGATTTAT-3′
ACOT2-1	5′-GCACTATATTGAGCCTCCTTA-3′
ACOT2-2	5′-CAGTTGTTCTCAGGTCTGAAT-3′
ACOT2-3	5′-GCTGTACCAATGGAGCCTGAA-3′
LYPA2-1	5′-GCATGAAATGAAGAACGGGAT-3′
LYPA2-2	5′-CCTCACGTCAAGTACATCTGT-3′
LYPA2-3	5′-CAGGGTCCAGTTCAAGACATA-3′
OTUB1-1	5′-AGGAGTATGCTGAAGATGACA-3′
OTUB1-2	5′-CACCACCAATCCGCACATCTT-3′
OTUB1-3	5′-TGTTTCTATCGGGCTTTCGGA-3′
OVCA2-1	5′-CCTCAAGTTCTTGGACCAGTT-3′
OVCA2-2	5′-CCAGATCAGACTTCGGGTC-3′
OVCA2-3	5′-CGGTTTATCCTCTTGGTGTCT-3′
PAFAH1B1-1	5′-GCAGATTATCTTCGTTCAAAT-3′
PAFAH1B1-2	5′-CGTATGGGATTACAAGAACAA-3′
PAFAH1B1-3	5′-GCTGAATTAGATGTGAATGAA-3′

Table 3. Primer sequences of qRT-PCR

Target	Sequence
GAPHD-F	5′-CCCACTCCTCCACCTTTGACG-3′
GAPD- R	5′-CACCACCCTGTTGCTGTAGCCA-3′
ABHD2-F	5′- CATGCTGGAGACTCCCGAAC -3′
ABHD2-R	5′- CAAACACCGGACGATCACGTA -3′
ACOT2-F	5′- CGTCCCGGCTGTACCAATG -3′
ACOT2-R	5′- GGAACCCTAATGATCTGACCAAC -3′
LYPA2-F	5′-CCCTCACGTCAAGTACATCTGT-3′
LYPA2-R	5′-GACGATTCGATTGGCAGGGAT-3′
OTUB1-F	5′-TCGGTCCTATACAAGGAGTATGC-3′
OTUB1-R	5′-GGTCTTGCGGATGTACGAGT-3′
OVCA2-F	5′-AGGAGGCCGACGTTTTCTC-3′
OVCA2-R	5′-AAGAGGATAAACCGTGGCAAG-3′
PAFAH1B1-F	5′-TCTTGGTCAGAAACGAGACCC-3′
PAFAH1B1-R	5′-GTGGTCGAATGAAATGTCCTGTA-3′
